# Compositional Shifts of Bacterial Communities Associated With *Pyropia yezoensis* and Surrounding Seawater Co-occurring With Red Rot Disease

**DOI:** 10.3389/fmicb.2019.01666

**Published:** 2019-07-23

**Authors:** Yong-Wei Yan, Hui-Chao Yang, Lei Tang, Jie Li, Yun-Xiang Mao, Zhao-Lan Mo

**Affiliations:** ^1^Laboratory for Marine Fisheries and Aquaculture, Qingdao National Laboratory for Marine Science and Technology, Key Laboratory of Maricultural Organism Disease Control, Ministry of Agriculture and Rural Affairs, Yellow Sea Fisheries Research Institute, Chinese Academy of Fishery Sciences, Qingdao, China; ^2^College of Fisheries and Life Science, Shanghai Ocean University, Shanghai, China; ^3^Key Laboratory of Marine Genetics and Breeding (Ocean University of China), Ministry of Education, Qingdao, China; ^4^Laboratory for Marine Biology and Biotechnology, Qingdao National Laboratory for Marine Science and Technology, Qingdao, China

**Keywords:** red rot disease, *Pyropia yezoensis*, seawater, bacterial communities, compositional shifts, potential indicator

## Abstract

*Pyropia yezoensis* is commercially the most important edible red alga in China, and red rot disease is viewed as one of the major constraints for its cultivation. Microbes within the oomycetic genus *Pythium* have been reported as the causative agents for this disease; however, little is known about the interactions between the disease and the epiphytic and planktonic bacterial communities. In the present study, bacterial communities associated with uninfected, locally infected, and seriously infected thalli collected from cultivation farms, and within seawater adjacent to the thalli, were investigated using in-depth 16S ribosomal RNA (rRNA) gene sequencing in conjunction with assessing multiple environmental factors. For both thalli and seawater, uninfected and infected communities were significantly different though alpha diversity was similar. Phylogenetic differences between epiphytic bacterial communities associated with *P. yezoensis* were mainly reflected by the relative changes in the dominant operational taxonomic units (OTUs) assigned as genus *Flavirhabdus*, genus *Sulfitobacter*, and family Rhodobacteraceae. The prevalent OTUs in seawater also differed in relative abundance across the communities and were affiliated with diverse taxa, including the phyla Actinobacteria, Verrucomicrobia, and Bacteroidetes, and the classes Alpha- and Gamma-proteobacteria. The differentiation of bacterial communities associated with *P. yezoensis* and seawater was primarily shaped by reactive silicate (RS) content and salinity, respectively. In particular, 14 potential indicators (two OTUs on *P. yezoensis* and twelve OTUs in seawater) were identified that significantly differentiated *P. yezoensis* health statuses and correlated with environmental changes. Overall, the present study provides insights into the alterations of bacterial communities associated with *P. yezoensis* and surrounding seawater co-occurring with red rot disease. Observed changes were closely associated with health status of algal host, and highlight the potential of using community differentiation to forecast disease occurrence.

## Introduction

The red algae *Pyropia* (Bangiales, Rhodophyta), also known as laver, is a globally popular food because of its high nutritional and medical value (Noda et al., 1990; MacArtain et al., 2007; Smith et al., 2010), and *Pyropia yezoensis* (Ueda) is a species of considerable commercial importance in East Asia. Being the largest producer of laver, China has an annual gross production worth almost US$1 billion^1^.

Economic incentives have driven intensive cultivation practices, and the increasing scale of production has caused a nutrient deficiency in the cultivate area, which affects the normal growth of *Pyropia* (Huang et al., 2000). *Pyropia* species have subsequently been rendered susceptible to several diseases, including red rot disease (Arasaki, 1947; Takahashi, 1977; Ding and Ma, 2005; Lee et al., 2017), *Olpidiopsis* disease (Arasaki, 1960; Ding and Ma, 2005; Kwak et al., 2017), and green spot disease (Fujita, 1990; Kim et al., 2016) in the blade stage, in addition to yellow spot disease (Guan et al., 2017) and white spot disease (Guan et al., 2013) in the shell-boring conchocelis stage. These diseases have caused a significant decline in *Pyropia* production in recent years, leading to heavy economic losses (Kim et al., 2014). In China, it has been estimated that disease outbreaks cause regular annual production losses of 25–30% at a regional scale (Gachon et al., 2010). Red rot disease has been reported as one of the major constraints in the profitable cultivation of *Pyropia* in China, Korea, and Japan, and no effective measurements have been developed to control the disease (Kim et al., 2014). Members of two species within the oomycetic genus *Pythium* (*P. porphyrae* and *P. chondricola*) are frequently reported as the causative agents of this disease (Lee et al., 2015, 2017; Diehl et al., 2017), although isolate of *Alternaria* is also capable of causing red rot disease (Mo et al., 2016). The pathogen *Pythium* can survive in both sea farms and terrestrial runoffs, even in the off-season, both of which could be the source of the zoospore inoculum initiating red-rot disease in farms (Ding and Ma, 2005; Kim et al., 2014; Klochkova et al., 2017). Under favored environments (e.g., high temperature, low salinity, and absence of free-change tide), pathogen zoospores adhere to the thalli and germinate on them, enabling the mycelium to grow through and kill the host cells (Ding and Ma, 2005). The infection spreads quickly in the farm and leads *P. yezoensis* rot to different degree, from red patches to numerous small holes on the blades, and then the whole cultivation net in a few days, by which time the disease can be observed (Ding and Ma, 2005; Kim et al., 2014).

The occurrence of algal disease originates from a complex interplay between host state, opportunistic pathogens and environmental factors (Egan et al., 2014). Normally, macroalgae are capable of attracting and favoring beneficial bacterial members to colonize and reproduce on their surface. These epiphytic bacteria, in turn, take part in the algal life cycle as well as aiding algae to fight against the colonization and infection of harmful microbes through secretion of secondary metabolites, and/or antimicrobials (Goecke et al., 2010). However, exposure to environmental stressors (e.g., high temperature) may affect the epiphytic bacterial assemblages directly or indirectly by weakening the host’s defensive capabilities, which consequently drives an abundance of surrounding pathogens on the host or causes commensals to turn virulent prior to physical signs of disease (Wahl et al., 2012; Egan and Gardiner, 2016). Correlative studies have repeatedly suggested structural shifts in associated bacterial communities co-occurring with an infection of the host, including corals (Bourne et al., 2008), sponges (Fan et al., 2013), and algae (Fernandes et al., 2012). For example, it has been demonstrated that temperature stress can cause a bacterial community shift toward a secretion reduction in defensive matter furanones, which results in enhanced infection by pathogens and ultimately bleaching disease in the red algae *Delisea pulchra* (Steinberg et al., 2011; Fernandes et al., 2012). Recently, multiple causative agents have been determined to be more abundant on bleached thalli. However, their coexistence on both unbleached and bleached thalli indicate their transition from commensals to virulent opportunistic pathogens (Zozaya-Valdes et al., 2015; Kumar et al., 2016). According to metagenomic data, multiple pathogens may function as a consortium to cause the bleaching events (Fernandes et al., 2012; Zozaya-Valdés et al., 2017). The aforementioned findings supported that the epiphytic bacterial assemblages correlate well with the health status of their algal hosts particularly prior to visible signs of infection.

Preliminary experiments have demonstrated that there is a change in microbiota composition co-occurring with the artificial infection of *P. yezoensis* using *P. porphyrae* spores (Khan et al., 2018). Unfortunately, this study lacked biological replicates and statistical analyses. In addition, multiple lines of evidence have demonstrated that high temperatures and low salinities are major factors which can induce *Pythium* to cause red rot disease (Ding and Ma, 2005; Klochkova et al., 2017), and those strong environmental pressures are exerted on *Pyropia* health prior to disease outbreaks. However, changes in the epiphytic bacterial communities associated with *Pyropia* in conjunction with assessments of health status and environmental conditions remain poorly understood. Specific information on the phylogeny of *Pyropia* microbiota is very important to improve our understanding of their interactions with red rot disease. Furthermore, bacterial assemblages in the surrounding seawater are not only sensitive to environmental changes, but can also be modified by the large amounts of organic matter and/or antimicrobial compounds released by macroalgae, or the dispersal of their epibiotic microbes, which are associated with host defensive capabilities (Goecke et al., 2010; Chen and Parfrey, 2018). Assessing bacterioplankton changes will provide us with a better understanding of the correlation between environmental changes and the occurrence of red rot disease, which may have important implications for cultivation management strategies.

The aims of the present research were: (i) to obtain a detailed view of the compositional shifts of *Pyropia*-associated and seawater-borne bacterial communities co-occurring with red rot disease happened in cultivation farms; and (ii) to screen bacterial taxa to be used as potential indicators so as to forecast the occurrence of red rot disease.

## Materials and Methods

### Sample Collection and Measurements of Physicochemical Parameters

**FIGURE 1:**
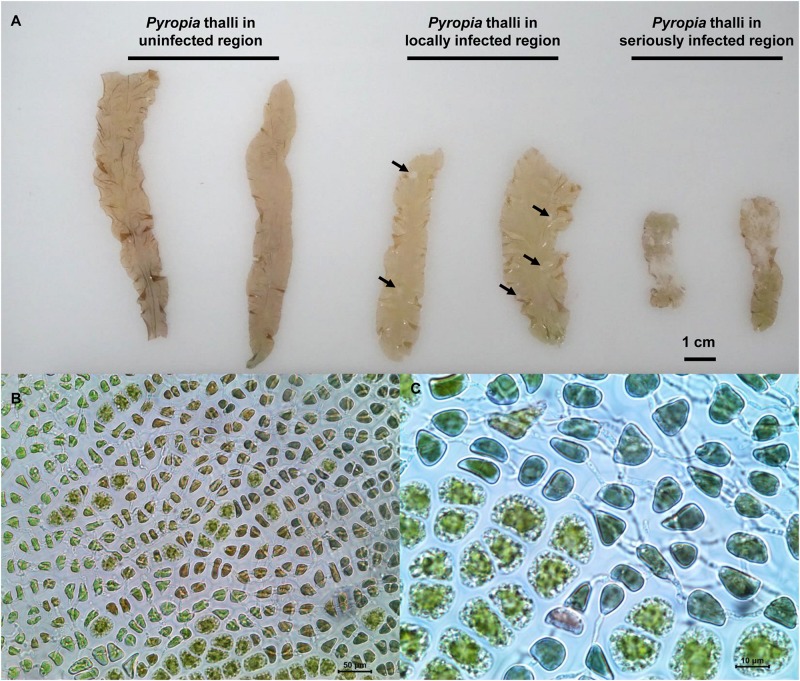
Comparison of different health-status *Pyropia yezoensis* thalli collected from three regions in the cultivation farms **(A)** and enlarged images of the thalli infected with *Pythium chondricola* **(B,C)**. The red rot section in the thallus is indicated by the arrowhead.

The *Pyropia yezoensis* incubation farms investigated in the present study were located in a coastal area of Xishu (34°45′N, 119°19′E), Haizhou Bay of Jiangsu Province of China, which is a typical *P. yezoensis* cultivation area with an annual aquaculture area over 7000 hectares. Contracted by many farmers, the area was divided into farms with each farmer evenly inoculated their own *Pyropia* genotype into their contracted farms for cultivation and management under natural environments. An outbreak of disease occurred in more than half of the area on January 22, 2018 with unknown reason. Based on the health status of *Pyropia* thalli, three regions (approximately 2–3 km apart) within farms (approximately 3 km^2^ in total) managed by the same farmer were selected for sampling, which included H (uninfected), D (locally infected), and DW (seriously infected) regions. *Pyropia* thalli in D region were characterized by 5–10 mm diameter spots compared with uninfected thalli (Figure 1A), while the thalli in DW region were characterized by spots spread across whole blades making the thalli almost completely white (Figure 1A). Through microscopic observation, cells of infected thalli had changed color from natural brown-red to violet-red or even green (Figure 1B), and mycelium penetration in the host cells was also observed (Figures 1B,C). All symptoms were highly consistent with morphological characteristics of red rot disease and a strain of *P. chondricola* was isolated and identified (data not shown).

For microbial community analysis, triplicate thalli samples and triplicate seawater samples from each region were collected, providing a total of 9 thalli samples, and 9 seawater samples. To make each of these biological replicates, adjacent triplicate samples (approximately 2 m apart) were also collected and pooled (thalli: hundreds of pieces in total; seawater: 3 L in total). All the samples were transported to the lab within 4 h and kept at 4°C, during which the thalli were submerged using *in situ* seawater. Thalli samples were washed three times with sterilized seawater to remove loosely associated microbes, and stored at −80°C until further processing. For each replicate of seawater, approximately 600 mL was pre-filtered through nylon mesh (200 μm pore size) to remove debris, and subsequently filtered through a 0.22 μm mixed cellulose membrane (MerckMillipore, Darmstadt, Germany) to collect microbial biomass.

Samples in the late stage of disease or those were decaying (DW samples in this study) were deemed inappropriate for the monitoring of pathogen infection because of the interference derived from saprophytic microbes (Egan and Gardiner, 2016). Therefore, environmental variables were measured using the triplicate seawater samples for both H and D regions, which were also used to evaluate environmental stress on bacterial assemblages. Seawater temperature, pH, salinity, and dissolved oxygen (DO) were recorded with appropriate sensors at a depth of 50 cm during the sampling process. The concentrations of ammonium, reactive silicate (RS), nitrite, nitrate, and phosphate were determined according to standard methods (Eaton et al., 2005), and three technical replicates were used to determine each variable for each sample.

### DNA Extraction

The Precellys^TM^-24 (Bertin Corp., Rockville, MD, United States) and a DNeasy PowerSoil^®^ Kit (Qiagen, Hilden, Germany) were used to extract the community DNA from thalli and seawater samples. Prior to extraction, samples were subjected to two times 6800 shakes min^−1^ for 30 s with an intermission of 5 min. DNA was stored at −20°C until amplification. DNA quality and integrity were assessed by gel electrophoresis.

### 16S rRNA Gene Amplification

For thalli samples, 16S rRNA genes were amplified using a two-round PCR protocol (Beckers et al., 2017), with the 5′-end of the second-round forward primer supplemented with a sample-specific 6-bp barcode for demultiplexing. For seawater samples, V4-V5 hypervariable regions of 16S rRNA genes were amplified using the primer pairs 515F and 926R (Walters et al., 2015), and a sample-specific 6-bp barcode was supplemented to the 5′ end of 515F. All PCR reactions were carried out with Q5 high-fidelity DNA polymerase (New England Biolabs, Ipswich, MA, United States). Each DNA sample was amplified in triplicate, and the products in correct length were purified using an AxyPrep DNA Gel Extraction Kit (Axygen, Tewksbury, MA, United States). The amplicon DNA concentrations were measured using a Quant-iT PicoGreen dsDNA Assay Kit with a BioTek FLx800 TBI reader (BioTek Instruments Inc., Winooski, VT, United States). Based on the quantification, the cleaned amplicons were mixed at equimolar ratios for library construction. Using a TruSeq DNA LT sample Prep Kit (Illumina, Boston, MA, United States), end-repairing, amplicon A-tailing, and adaptor ligating were carried out according to standard protocols. After ligation of the sequencing adaptors, the composite library was cleaned again using an AxyPrep DNA Gel Extraction Kit (Axygen, Tewksbury, MA, United States). Next-generation sequencing (NGS) was performed on an Illumina MiSeq platform using the 2 × 250 protocol.

### Bioinformatic Analysis

Sequences were pre-processed using windows-compatible 32-bit USEARCH software v10.0.240 (Edgar, 2010). Paired-end reads were merged using the command “fastq_mergepairs.” After primer truncation, merged reads were quality filtered by setting a maximum number of expected errors (maxee 1.0) using the command “fastq_filter.” Operational taxonomic unit (OTU) clustering (97% identity) were performed using USEARCH according to standard protocol, and singletons and chimeric reads were removed from further analysis. In QIIME v1.9.0 (Caporaso et al., 2010), taxonomic information was assigned to each representative OTU sequence using the ribosomal database project (RDP) classifier (Wang et al., 2007), and with a minimum bootstrap confidence of 80% against the SILVA_132_QIIME_release database^2^. OTUs identified as unassigned, chloroplast, or mitochondria were discarded. The alpha diversity indices of the datasets were calculated in QIIME based on the filtered OTUs using the command “alpha_rarefaction.py,” which involved Good’s coverage, Observed species, Chao1, Shannon, and phylogenetic distance (PD). To determine the amount of diversity shared between two communities (beta diversity), Bray-Curtis distances were calculated between all pairs of samples using the command “beta_diversity_through_plots.py.”

### Statistical Analysis

An unpaired *t*-test was used to evaluate the differences in seawater variables (H region vs. D region). An unpaired wilcoxon rank sum test was used to evaluate alpha diversity indices between bacterial communities in R (R Core Team, 2009). Non-metric multi-dimensional scaling (NMDS) ordination was performed to evaluate the differences in microbial community structures based on Bray-Curtis distances using the “vegan” package in R (R Core Team, 2009). Bray-Curtis distance based dissimilarity tests (e.g., MRPP, ANOSIM, and Adonis) were employed to test the significance of the differences among the bacterial community compositions in QIIME using the command “compare_categories.py.”

To identify the bacterial assemblages that distinguished *P. yezoensis* health status, the taxa were screened at the OTU level through comparison of the relative abundance in the H and D communities, similar to the identification of shrimp indicators (Xiong et al., 2014; Zhang et al., 2014). To make inter-group differences as great as possible, status-related OTUs were selected that: (1) were contributed to differentiate H and D datasets and had an arithmetic average difference of >0.4% relative abundance; (2) were significantly associated with *P. yezoensis* health status (*P* < 0.05). A similarity percentage analysis (SIMPER) was applied to identify the OTUs that were primarily responsible for observed differences between datasets based on PAST software v3.1.7 (Hammer et al., 2001). An unpaired wilcoxon rank sum test was used to evaluate the significant differences in bacterial abundance between bacterial communities in R (R Core Team, 2009). A redundancy analysis (RDA) with 999 Monte Carlo permutation test was implemented to reveal the relationships between bacterial assemblages and environmental variables in R using the “vegan” package (R Core Team, 2009). Pearson’s correlation coefficients between the health status-related OTUs and the environmental variables were calculated using the “Hmisc” package in R (R Core Team, 2009).

### Nucleotide Sequence Accession Numbers

High-throughput raw sequence data have been deposited in the US national center for biotechnology information (NCBI) GenBank short read archive (SRA) under the accession numbers SRR8284879–SRR8284896.

## Results

### Physico-Chemical Characteristics of Seawater

The physico-chemical characteristics of seawater samples are summarized in Table 1. Most environmental parameters were relatively stable within the regions, and no significant differences were observed except in salinity and RS concentration. Salinity was significantly different between the H (27.04 ± 0.04%) and D regions (26.37 ± 0.06%) (*P* < 0.001), as were RS concentrations (*P* = 0.002). For both parameters, lower values were found in the D region.

**TABLE 1:** Summary of the seawater sample physico-chemical parameters for the H and D regions.

	**H**	**D**	***P***
Temperature (°C)	3.83 ± 0.06	3.83 ± 0.06	0.500
Dissolved oxygen (%)	125.80 ± 6.63	131.80 ± 11.46	0.245
pH	8.21 ± 0.01	8.20 ± 0.09	0.406
Salinity (‰)	27.04 ± 0.04	26.37 ± 0.06	**<0.001**
Reactive silicate (μmol/L)	7.94 ± 1.21	1.66 ± 0.51	**0.002**
Phosphate (μmol/L)	0.25 ± 0.02	0.25 ± 0.03	0.500
Nitrite (μmol/L)	2.85 ± 0.16	2.80 ± 0.04	0.322
Nitrate (μmol/L)	25.20 ± 2.99	22.80 ± 3.33	0.203
Ammonium (μmol/L)	0.76 ± 0.52	0.43 ± 0.51	0.241

*The data represent the mean ± standard deviation, *n* = 3. Bold *P* values indicate significant differences between the two health status categories. Means were compared using an unpaired *t*-test and significance was determined as *P* < 0.05.*

### Bacterial Community Diversities

After quality control, a total of 520,061 high-quality sequences and 23,631–36,411 sequences per sample (mean ± standard deviation = 28892 ± 3650) were obtained. More than 38% (52.46 ± 12.14%) of the sequences were able to be assigned as bacteria (14922 ± 2737), and more than 99% of the bacterial reads could be classified at the phylum level. A total of 65 and 336 bacterial OTUs were obtained for *P. yezoensis* and seawater samples, respectively, which could be assigned into 5 and 17 phyla, respectively, according to the SILVA database. From the demonstrations of venndiagram, most (approximately 60%) of the bacterial OTUs associated with *P. yezoensis* were shared by the three groups of datasets (39/65), as was the case for seawater also (203/336) (Supplementary Figure S1).

**FIGURE 2:**
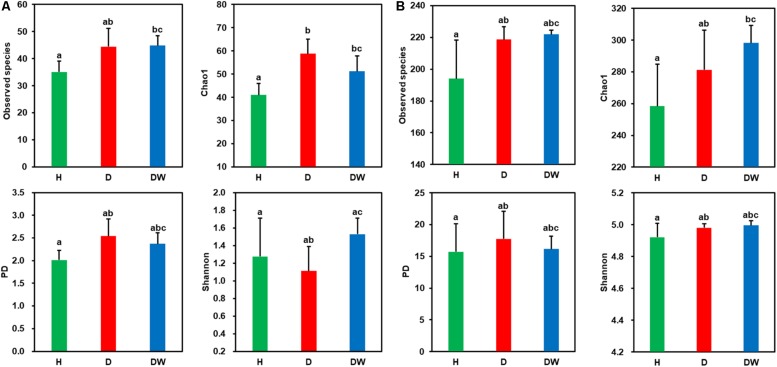
Comparisons of diversity indices for *Pyropia yezoensis*-associated **(A)** and seawater-borne **(B)** bacterial communities between different health statuses. Error bars indicate standard deviations, and the letters on the error bars indicate significant differences (*P* < 0.05) based on unpaired wilcoxon rank sum tests.

Good’s coverage for all samples was greater than 94% (Supplementary Figure S2A), indicating that sequencing captured the majority of the bacterial diversity in the samples. Ten-thousand randomly selected sequences (the lowest number in samples was 10,924) were used to estimate alpha diversity (i.e., Observed species, Chao1, PD, and Shannon) of each dataset. These alpha diversity indices indicated that seawater held much higher (4–7 times greater) bacterial diversity than *P. yezoensis* (Figure 2 and Supplementary Figure S2). However, none of the diversity indices, for both *P. yezoensis* (Figure 2A) and seawater (Figure 2B), displayed a significant increase across the health statuses. On the one hand, diversity was not significantly different between the H and D communities from the demonstrations of the four indices for both *P. yezoensis* and seawater, though *P. yezoensis* in the D region had significantly higher Chao1 values than those in the H region. On the other hand, all the DW communities held similar diversity indices compared with D communities except *P. yezoensis*-associated Shannon index. In addition, alpha diversity indices did not display significant differences between the H and DW communities, though some values varied.

**FIGURE 3:**
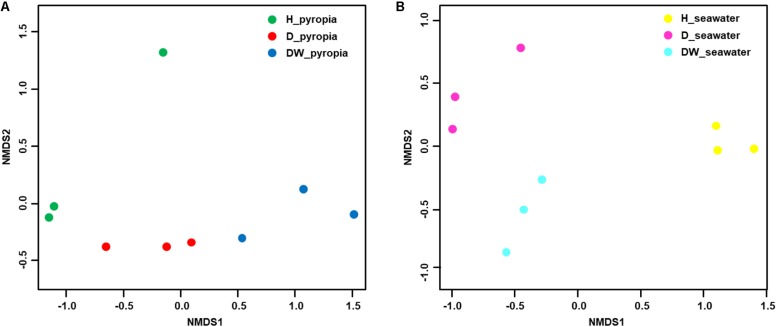
Relationships between individual datasets illustrated by non-metric multi-dimensional scaling (NMDS) ordination. **(A)** *Pyropia yezoensis*-associated community datasets. **(B)** Seawater-associated community datasets.

In order to further interpret the diversities, a NMDS ordination based on Bray-Curtis distances was performed to elucidate the clustering relationship between the bacterial communities (Figure 3). For both *P. yezoensis* (Figure 3A) and seawater (Figure 3B), the bacterial communities were clearly clustered in accordance with *P. yezoensis* health status which included H, D, and DW samples. Bray-Curtis distance-based ANOSIM, Adonis, and MRPP analyses also confirmed a significant difference among the three groups for both *P. yezoensis*- and seawater-associated bacterial communities (Table 2).

**TABLE 2:** Significance tests of the differences in *Pyropia yezoensis*- and seawater-associated bacterial community structures, respectively on the basis of Bray-Curtis dissimilarities.

**Communities**	**ANOSIM**		**Adonis**		**MRPP**	
	
	***R***	***P***	***R*^2^**	***P***	***A***	***P***
H vs. D vs. DW (*Pyropia*)	0.646	**0.003**	0.670	**0.004**	0.347	**0.003**
H vs. D vs. DW (Seawater)	1.000	**0.007**	0.788	**0.001**	0.444	**0.006**

*Bold *P* values indicate significant differences between health statuses.*

### Bacterial Community Structures

**FIGURE 4:**
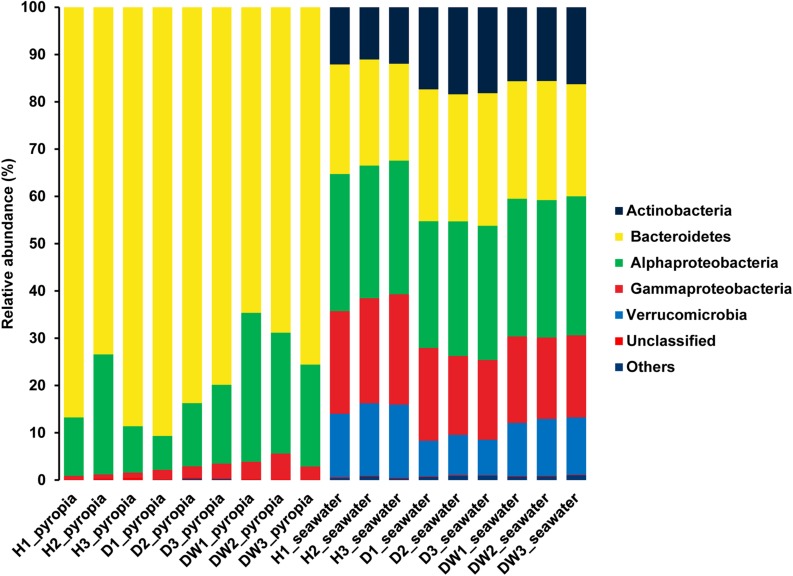
Bacterial community structures associated with *Pyropia yezoensis* and seawater at the phylum/class level. H, uninfected; D, locally infected; DW, seriously infected.

Two taxa predominated within the *P. yezoensis*-associated bacterial communities: Bacteroidetes (79.09 ± 9.10%) and Alphaproteobacteria (18.15 ± 8.25%) (Figure 4). At the OTU level, all the datasets were predominated by OTU1 affiliated with the genus *Flavirhabdus* (76.78 ± 9.70%) within Bacteroidetes. Relative abundance decreased from 78.95 ± 10.97% in H region *P. yezoensis* and 83.36 ± 5.73% in D region *P. yezoensis* to 68.04 ± 6.06% in DW region *P. yezoensis* (Supplementary Figure S3A). The following two prevalent OTUs both belonged to Alphaproteobacteria, with OTU4 (*Sulfitobacter*, 11.64 ± 9.22%) increasing (3.32 ± 1.90%, 9.11 ± 4.08%, and 22.50 ± 5.39%) but OTU5 (Rhodobacteraceae, 3.94 ± 5.88%) decreasing (10.33 ± 6.80%, 1.08 ± 0.12%, and 0.42 ± 0.10%) across H, D, and DW datasets, respectively (Supplementary Figure S3A). These OTUs also contributed 15.87% dissimilarity to differentiate the bacterial communities between different health statuses according to SIMPER analysis (Supplementary Table S1).

Bacterial communities of seawater displayed different profiles compared with *P. yezoensis*-associated communities. The dominant taxa in the seawater were affiliated with Bacteroidetes (24.73 ± 2.55%) and Alphaproteobacteria (28.51 ± 0.77%), followed by Gammaproteobacteria (19.25 ± 2.58%), Actinobacteria (15.18 ± 2.80%), and Verrucomicrobia (11.47 ± 3.06%) (Figure 4). The prevalent OTUs across all the datasets were also distributed within all these taxa, such as OTU1 (“*Candidatus* Aquiluna”) in Actinobacteria, OTU5 (*Marivita*), OTU9 (SAR11 clade Ia), and OTU7 (*Planktomarina*) in Alphaproteobacteria, OTU2 (SAR92 clade) in Gammaproteobacteria, OTU4 (*Persicirhabdus*) and OTU8 (*Lentimonas*) in Verrucomicrobia, and OTU10 (*Loktanella*) in Bacteroidetes (Supplementary Figure S3B). The relative abundance of OTU2, OTU9, and OTU8 in H region seawater datasets were relatively higher than that in D and DW datasets, in contrast to the situation of OTU1, OTU5, OTU7, and OTU10. The relative abundance of OTU4 in H and D region seawater samples was relatively lower than that of DW region seawater (Supplementary Figure S3B). The SIMPER analysis revealed that all these OTUs contributed to nearly 10% dissimilarity to differentiate each dataset (Supplementary Table S2).

### Identification of Status-Related Bacterial Taxa

**FIGURE 5:**
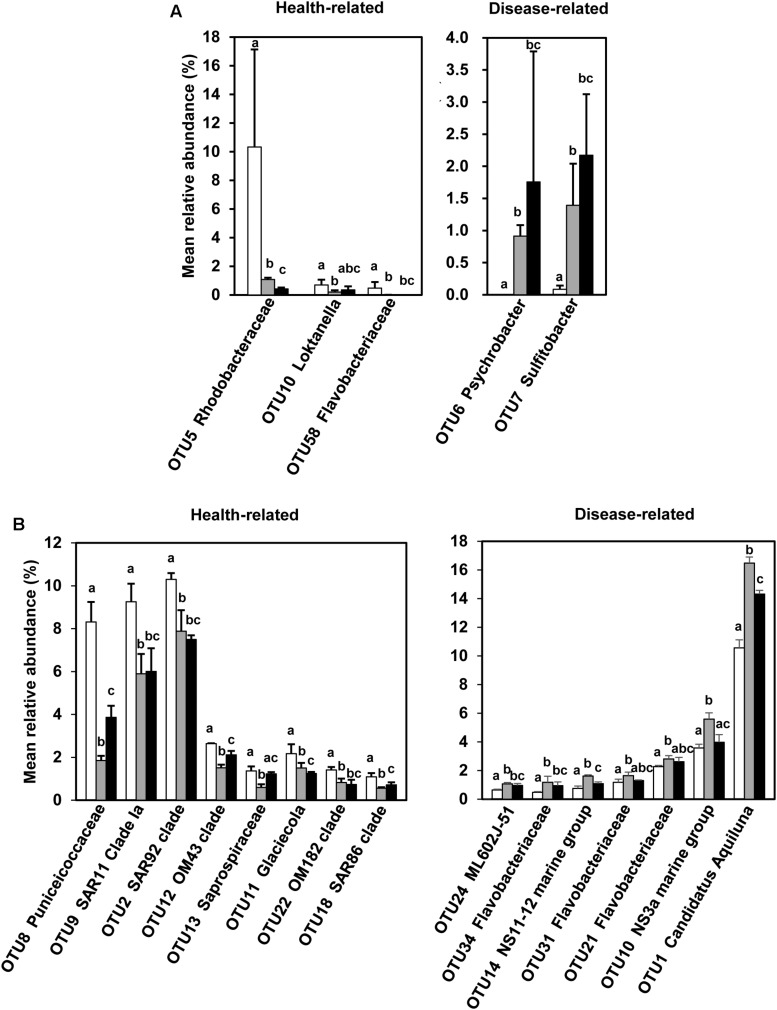
Screened health-status related bacterial OTUs and the comparisons of their relative abundance across the three groups. **(A)** Screened bacterial OTUs associated with *Pyropia yezoensis*. **(B)** Screened bacterial OTUs associated with seawater. White, gray, and black bars represent relative abundances of bacterial OTUs in the H, D, and DW datasets, respectively. The error bars indicate standard deviations, and the letters on the error bars indicate significant differences (*P* < 0.05) based on unpaired wilcoxon rank sum tests.

Using two basic principles, this procedure identified 5 and 15 OTUs for *P. yezoensis* (Figure 5A) and seawater (Figure 5B), respectively. The relative abundance ranged from 0.00 ± 0.00% to 0.69 ± 0.37% and from 0.00 ± 0.00% to 1.39 ± 0.65% in H and D region *P. yezoensis* datasets, respectively. In contrast, relative abundance ranged from 0.48 ± 0.05% to 10.56 ± 0.56% and from 0.56 ± 0.05% to 16.48 ± 0.42% in H and D region seawater datasets, respectively.

For *P. yezoensis*, the relative abundance of associated bacterial OTUs belonging to the genus *Psychrobacter* (OTU6, class Gammaproteobacteria) and *Sulfitobacter* (OTU7, class Alphaproteobacteria) were significantly increased in the D communities compared with those in H. In contrast, OTUs affiliated with the families Rhodobacteraceae (OTU5, class Alphaproteobacteria) and Flavobacteriaceae (OTU58, phylum Bacteroidetes) and the genus *Loktanella* (OTU10, class Alphaproteobacteria) were more enriched in the H datasets compared with the D datasets. In the seawater samples, seven OTUs were more abundant in the seawater in which disease occurred, with four belonging to the family Flavobacteriaceae (OTU10, OTU21, OTU31, and OTU34) and the remaining three belonging to ML602J-51 within family Microbacteriaceae (OTU24, phylum Actinobacteria), NS11-22 marine group (OTU14, phylum Bacteroidetes), and the genus “*Ca.* Aquiluna” (OTU1, phylum Actinobacteria), respectively. Most of the more abundant OTUs in seawater in which no-infection was present were classified as belonging to the class Gammaproteobacteria (OTU2, OTU12, OTU11, OTU22, and OTU18), with the rest assigned to the genus *Letimonas* (OTU8, phylum Verrucomicrobia), Clade Ia of SAR11 (OTU9, class Alphaproteobacteria), and family Saprospiraceae (OTU13, phylum Bacteroidetes).

In DW *P. yezoensis* datasets, the relative abundance of all the OTUs, except OTU5, were not significantly different from those in D region *P. yezoensis*. However, the situation was more complicated with respect to the seawater in which they grew. For example, the relative abundance of disease-related OTU1, OTU10, and OTU14 in DW seawater was significantly lower than those in D seawater, while abundances of OTU21, OTU31, OTU34, and OTU24 were not statistically different between the two types of seawater datasets. The relative abundances of some of the health-related OTUs (e.g., OTU8, OTU12, OTU13, and OTU18) in DW seawater were significantly greater when compared with D seawater.

### Linking Bacterial Community Structures to Seawater Properties

**FIGURE 6:**
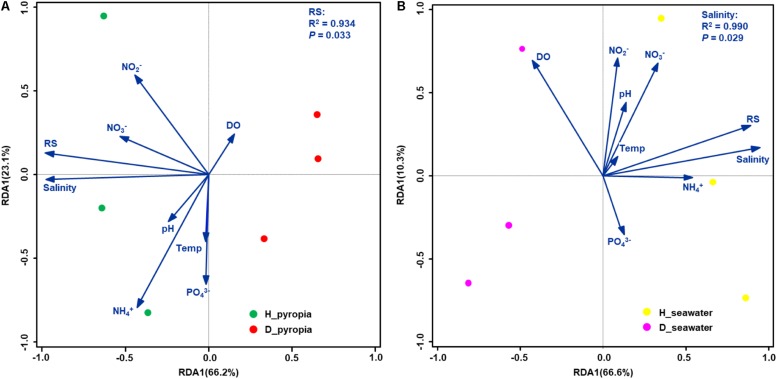
Biplots of redundancy analysis (RDA) for bacterial communities and environmental parameters. Community datasets associated with *Pyropia yezoensis* **(A)** and seawater **(B)** were used. Temp, temperature; DO, dissolved oxygen; RS, reactive silicate.

According to the RDA results, the concentrations of ammonium, nitrite, nitrate and phosphate, in addition to temperature, pH, and DO, had no significant effects on the distribution between H and D bacterial communities (*P* > 0.05, Monte Carlo test) (Figure 6). RS concentration (*P* = 0.033) and salinity (*P* = 0.029) were significantly correlated with *P. yezoensis*-associated and seawater-borne bacterial communities, respectively (Figure 6). Specifically, bacterial communities associated with uninfected *P. yezoensis* were positively correlated with the RS concentration (Figure 6A), and bacterial communities within the seawater such *P. yezoensis* grew in were positively correlated with salinity (Figure 6B).

Pearson’s correlation coefficients between the health status-related OTUs and the environmental variables were also examined (Table 3). For *P. yezoensis*, only disease-related bacterial OTUs were both significantly correlated with the salinity and RS concentration. With respect to seawater, all except OTU11, OTU34, and OTU31 were significantly correlated with these two parameters.

**TABLE 3:** Pearson’s correlation coefficients between health-status related bacterial taxa and environmental parameters.

**Taxonomic information**	**Salinity**		**Reactive silicate**	
	
	**R**	**P**	**R**	**P**
***Pyropia yezoensis***				
OTU5 Alphaproteobacteria; Rhodobacterales; Rhodobacteraceae	0.79	0.060	0.88	**0.022**
OTU10 Alphaproteobacteria; Rhodobacterales; Rhodobacteraceae; Loktanella	0.70	0.125	0.73	0.097
OTU58 Bacteroidetes; Bacteroidia; Flavobacteriales; Flavobacteriaceae	0.69	0.132	0.77	0.074
OTU6 Gammaproteobacteria; Pseudomonadales; Moraxellaceae; Psychrobacter	–0.99	**0.000**	–0.97	**0.002**
OTU7 Alphaproteobacteria; Rhodobacterales; Rhodobacteraceae; Sulfitobacter	–0.91	**0.013**	–0.88	**0.021**
**Seawater**				
OTU8 Verrucomicrobia; Verrucomicrobiae; Opitutales; Puniceicoccaceae; Lentimonas	0.98	**0.000**	0.95	**0.004**
OTU9 Alphaproteobacteria; SAR11 clade; Clade I; Clade Ia	0.92	**0.010**	0.87	**0.023**
OTU2 Gammaproteobacteria; Cellvibrionales; Porticoccaceae; SAR92 clade	0.92	**0.008**	0.89	**0.019**
OTU12 Gammaproteobacteria; Betaproteobacteriales; Methylophilaceae; OM43 clade	0.99	**0.000**	0.97	**0.002**
OTU13 Bacteroidetes; Bacteroidia; Chitinophagales; Saprospiraceae; Uncultured	0.92	**0.008**	0.95	**0.004**
OTU11 Gammaproteobacteria; Alteromonadales; Alteromonadaceae; Glaciecola	0.79	0.060	0.71	0.115
OTU22 Gammaproteobacteria; OM182 clade	0.91	**0.013**	0.86	**0.030**
OTU18 Gammaproteobacteria; SAR86 clade	0.95	**0.004**	0.90	**0.014**
Otu24 Actinobacteria; Actinobacteria; Micrococcales; Microbacteriaceae; ML602J-51	–0.98	**0.001**	–0.96	**0.003**
OTU34 Bacteroidetes; Bacteroidia; Flavobacteriales; Flavobacteriaceae	–0.80	0.057	–0.78	0.066
OTU14 Bacteroidetes; Bacteroidia; Sphingobacteriales; NS11-12 marine group	–0.96	**0.003**	–0.91	**0.012**
Otu31 Bacteroidetes; Bacteroidia; Flavobacteriales; Flavobacteriaceae	–0.79	0.063	–0.70	0.120
OTU21 Bacteroidetes; Bacteroidia; Flavobacteriales; Flavobacteriaceae	–0.87	**0.024**	–0.96	**0.034**
OTU10 Bacteroidetes; Bacteroidia; Flavobacteriales; Flavobacteriaceae; NS3a marine group	–0.94	**0.006**	–0.89	**0.016**
OTU1 Actinobacteria; Actinobacteria; Micrococcales; Microbacteriaceae; Candidatus Aquiluna	–1.00	**0.000**	–0.99	**0.000**

*Bold *P* values indicate significant correlations (*P* < 0.05).*

## Discussion

Although red rot disease is usually caused by oomycetic members within the genus *Pythium*, compositional shifts in the microbiota may co-occur with the infection (Khan et al., 2018). However, due to poor replication and limited statistical analyses in previous studies, information regarding the composition of the microbiota on uninfected and infected *P. yezoensis* thalli is still very limited. This knowledge gap currently hinders us from revealing the underlying interactions between the occurrence of red rot disease and epiphytic bacterial communities. In addition, scarce bacterioplankton data have been provided during the occurrence of red rot disease, though environmental conditions (e.g., temperature and salinity) are known to be correlated with the disease (Ding and Ma, 2005; Klochkova et al., 2017). In the present study, we provide insights into the compositional shifts of *P. yezoensis*-associated bacterial communities co-occurring with the outbreak of red rot disease, and the alterations of bacterial communities in the surrounding water column.

### Bacterial Community Diversities

The community profiles of both *P. yezoensis* and seawater were similar to each other even when health statuses of the relative thalli were different. This result was also supported by the large fraction of shared OTUs. However, NMDS and dissimilarity tests demonstrated the differences in bacterial communities among the different health statuses for both *P. yezoensis* and seawater, although their alpha-diversity indices were not significantly different. Therefore, changes were largely driven by substantial shifts in the abundance of a few bacterial taxa. For example, high standard deviations were observed for all the major bacterial taxa associated with *P. yezoensis*, which suggests profound alterations in bacterial composition. Small standard deviations were observed for seawater-borne microbes, which possibly suggests several bacterial OTUs were more sensitive to the variations of physico-chemical changes of seawater (e.g., salinity and RS), and which may function as driving factors for disease occurrence in the present study.

### Bacterial Community Structure Shifts

In the present study, the epiphytic bacterial communities on all the sampled *P. yezoensis* thalli were dominated by Alphaproteobacteria and Bacteroidetes, as determined using NGS, which is consistent with those associated with the green alga *Ulva australis* (Burke et al., 2011), and the red algae *Porphyra umbilicalis* (Miranda et al., 2013) and *D. pulchra* (Zozaya-Valdes et al., 2015). Such dominance possibly suggests the prevalence of these two phyla on the surface of macroalgae. The predominant genus *Flavirhabdus* within Bacteroidetes (OTU1 in this study) was proposed as genus *Lacinutrix* recently (Nedashkovskaya et al., 2016), and members of *Lacinutrix* have been recovered from other algal species as common inhabitants (Nedashkovskaya et al., 2008, 2016; Huang et al., 2016). Genomic information has proved that they have evolved to reduce surrounding sulfate into sulfide (i.e., H_2_S) (Lee et al., 2016). As is also the case for macroalgae, the rapid assimilation of such gas in plant quickly causes the accumulation of reactive oxygen species and oxidative stress, so as to be cytotoxical to pathogens, and activate further defense reactions (Calderwood and Kopriva, 2014; Egan et al., 2014; Parveen et al., 2017). The significant role of bacteria in the family Rhodobacteraceae within Alphaproteobacteria (prevalent OTU5 in this study) derives mostly from *Roseobacter* group since 70 of 100 genera within the family are affiliated with this group (Dogs et al., 2017). Although metabolically diverse, members within this group may inhibit settlement of fouling organisms on algae or function as probionts that deter algal pathogens, in addition to secret Vitamin B12 for algae growth (Luo and Moran, 2014; Dogs et al., 2017). Therefore, the notable reduction in abundance in D *P. yezoensis* and/or DW *P. yezoensis* datasets for both OTUs indicates the defensive capability to the host, relying on the epiphytic bacteria, decreased notably with infection. In contrast, bacterial strains in the genus *Sulfitobacter* within Alphaproteobacteria (OTU4 in this study) are reportedly capable of consuming sulfated polysaccharide in addition to sulfur oxidation (Dogs et al., 2017), and have been recovered from several algal species including *P. yezoensis* (Kanagasabhapathy et al., 2008; Fukui et al., 2014; Wu et al., 2014; Albakosh et al., 2016; Beleneva et al., 2017). As highly enriched in porphyran for *P. yezoensis*, sulfated polysaccharide degradation of such structural elements, cell walls, or storage materials can have a detrimental impact on the macroalgal host. Therefore, the increased abundance of *Sulfitobacter* on infected *P. yezoensis* may indicate its synergistic effect on the infection of *P. chondricola* co-occurring with the decrease of host health status.

Evidence indicates that compositional shifts in microbial community structure can substantially alter ecological function (Kritzberg et al., 2006; Comte and Del Giorgio, 2010; Teeling et al., 2012). For example, NS3a group and SAR11 are distributed to a greater extent in accordance with changes in the environment, and thus contribute more strongly to determining overall biogeographical patterns of marine bacterial communities (Lindh et al., 2016). In our study, bacterial communities within seawater also experienced community structure shifts, which were notably revealed by the abundance alterations of prevalent OTUs (e.g., “*Ca.* Aquiluna,” *Marivita*, and SAR92 clade) that were distributed within the dominant phylum/class. The majority of these bacterial members have also been discovered in the bacterioplankton of coastal seawater (Stingl et al., 2007; Hwang et al., 2009; Freitas et al., 2012; Giebel et al., 2013; Shan et al., 2015; Teeling et al., 2016), and are mainly involved in the nutrient cycles rather than pathogenicity, such as acquisition of nitrogen and sulfur, degradation of organic matter, and utilization of small organic molecules (Arrigo, 2004; Stingl et al., 2007; Teeling et al., 2012, 2016). Although certain taxa are less understood (e.g., the *Lentimonas* genus), the close relationship between the changes in bacterioplankton composition and disease occurrence lead us to hypothesize that the compositional shifts in the microbiota could indicate the health status of *P. yezoensis*.

### Health-Status Related Bacterial Taxa

It is possible for saprophytic microbes to obscure the identity of the true primary pathogens in the late stages of disease, and thus make it more complicated to determine early stage infections (Egan and Gardiner, 2016). Considering this, and the fact that the DW datasets did not usually display steadily increasing or decreasing trends across the health statuses, DW datasets were not used to identify health status related bacterial taxa for both *P. yezoensis* and seawater.

Statistically, three OTUs in total, including OTU10 (*Loktanella* in Rhodobacteraceae) and OTU58 (Flavobacteriaceae) and also the prevalent OTU5 (Rhodobacteraceae), were shown to account for significantly higher abundance on uninfected *P. yezoensis* thalli. Like some other bacteria within the family Rhodobacteraceae, bacteria in the genus *Loktanella* also have the capacity to stimulate algal growth through vitamin secretion (Dogs et al., 2017). Bacteria in this genus also have algicidal activity (Bloh et al., 2016), suggesting that the colonization of harmful algae onto *P. yezoensis* may have been inhibited. In addition, bacteria in the family Flavobacteriaceae are reportedly capable of releasing morphogens, and thus induce morphogenesis of macroalgae such as *U. mutabilis* (Weiss et al., 2017). Therefore, the three OTUs on *P. yezoensis* were more indicative for the uninfected status of *P. yezoensis*. In contrast, OTU6 and OTU7, affiliated with the genera *Psychrobacter* and *Sulfitobacter*, respectively, were significantly correlated with both the infected status of *P. yezoensis* and deteriorated water quality (salinity and RS). Recently, strains of *Psychrobacter* have been isolated from the surface of normal *P. yezoensis* and several other macroalgae (Kanagasabhapathy et al., 2008; Fukui et al., 2014; Wu et al., 2014; Albakosh et al., 2016; Beleneva et al., 2017). However, the appearance of *Psychrobacter* on the thalli of *P. yezoensis* likely indicate a synergistic mechanism for the infection by *Pythium* spp. due to potential degradation of the cell wall (Kim et al., 2011; Lin et al., 2017) and antibiotic matter (e.g., phenol) (Moghadam et al., 2016).

A total of 15 planktonic bacterial OTUs, distributed within diverse bacterial taxa, were significantly correlated with *P. yezoensis* health status, including the prevalent OTUs in the water-borne bacterial communities (i.e., OTU8, OTU9, OTU2, OTU10, and OTU1). Like the prevalent OTUs in seawater, most of the status-related planktonic bacterial taxa (e.g., OM43 clade, *Glaciecola*, SAR86 clade, Flavobacteriaceae, and NS11-12 marine group) have also been frequently discovered in coastal seawater (Na et al., 2011; Shan et al., 2015; Lindh et al., 2016; Teeling et al., 2016), and indicate the diversely dynamic ecological functions in the nutrient cycles within this coastal aquaculture area. In the area of aquaculture, it has been documented that planktonic bacteria with significant abundance alterations in seawater can well indicate the health status of cultured shrimps (Xiong et al., 2014; Zhang et al., 2014). As closely connected with the macroalgae, it is reasonable to deduce that the microbial assemblages that preferentially occur in infected or uninfected water bodies may also serve as diagnostic indicators.

However, not all the aforementioned screened taxa could be used as potential indicators to forecast health status of *P. yezoensis*, because environmental stress is also a factor that should be carefully considered. Red rot disease usually occurs within sea areas where *P. yezoensis* is cultivated in considerably higher densities. In this context, a water body is less capable of efficient exchange, leading to environmental stress (e.g., a shortage of nutritive salt supply), deterioration in the physical condition of algae and a reduction in the beneficial effects from epibacterial communities. Consequently, high densities cultivation areas provide many more opportunities for the adherence of *Pythium* spores on *P. yezoensis* thalli which contributes to infection. The reduced salinity and RS concentrations in the D region coupled with bacterial community shifts on the thalli highly supports this observation. Low salinity areas are suitable for the growth and infection of *Pythium* (Ding and Ma, 2005; Klochkova et al., 2017) and the role of RS in the growth of macroalgae is somewhat established (Mehdipour et al., 2015; Haroon et al., 2018), although a direct relationship between RS, and the physical status of *P. yezoensis* has been poorly revealed. Taken together, the bacterial taxa that significantly correlated with both salinity and RS, which included two OTUs (OTU6 and OTU7) on *P. yezoensis* and twelve OTUs (OTU8, OTU9, OTU2, OTU12, OTU13, OTU22, OTU18, OTU24, OTU14, OTU21, OTU10, and OTU1) in seawater, are likely to be more reliable potential health-status indicators for *P. yezoensis*.

## Conclusion

In the present study, an outbreak of red rot disease was reported in an aquaculture area in Jiangsu Province, China. Although different health statuses of *P. yezoensis* thalli shared most of the detected OTUs and occupied similar alpha diversity indices, the alterations of epiphytic microbial community composition are associated with *Pyropia* health status. This was also the case for the bacterial communities within the seawater in which *P. yezoensis* was cultivated. RS content and salinity were major environmental factors that significantly shaped the differentiation of bacterial communities associated with *P. yezoensis* and seawater, respectively. Differences in bacterial taxa on the thalli indicate their potential roles in algal defensive capabilities or a synergistic effect on the infection by pathogens, whereas those in the seawater indicate, and altered ecological functions in nutrient cycles correlated with infection. Overall, results broaden the understanding of the relationships between the occurrence of red rot disease and the microbiota associated with *P. yezoensis* and seawater. Two OTUs derived from *P. yezoensis* and twelve OTUs derived from seawater could be potential indicators to evaluate the health status of *P. yezoensis* and thus to predict the occurrence of red rot disease. Nevertheless, more replicates are required to analyze both the bacterial communities and physiological index of *P. yezoensis* in the course of disease so as to figure out how the relative abundance of indicative taxa will precisely forecast disease outbreak. As another important factor for red rot disease, temperature should also be taken into consideration in future study.

## Author Contributions

Y-WY, Y-XM, and Z-LM designed the experiments and prepared the manuscript. Y-WY, H-CY, LT, and JL conducted the experiments. Y-WY carried out the microbial analysis. All authors were involved in revision of the manuscript and approved its final version.

## Conflict of Interest Statement

The authors declare that the research was conducted in the absence of any commercial or financial relationships that could be construed as a potential conflict of interest.

## Footnotes

1 http://www.fao.org/fishery/statistics/global-aquaculture-production/query/en

2 https://www.arb-silva.de/no_cache/download/archive/qiime/

## Supplementary Material

FIGURE S1 Venn diagrams showing the total number of OTUs and the number of shared OTUs across all *Pyropia yezoensis* **(A)** and seawater **(B)** datasets.

FIGURE S2 Rarefaction curves of *Pyropia yezoensis* and seawater datasets by **(A)** Good’s coverage, **(B)** Observed species, **(C)** Chao1, **(D)** PD, and **(E)** Shannon with OTU being defined at 97% sequence similarity.

FIGURE S3 Distribution of the most abundant 30 OTUs across the *Pyropia yezoensis* **(A)** and seawater **(B)** datasets. Colors represent the relative abundance of each OTU.

TABLE S1 Dissimilarity contribution of bacterial OTUs within *Pyropia yezoensis* datasets according to SIMPER analysis.

TABLE S2 Dissimilarity contribution of bacterial OTUs within seawater datasets according to SIMPER analysis.
