# Supplementary figures and images for: Compositional Shifts of Bacterial Communities Associated With Pyropia yezoensis and Surrounding Seawater Co-occurring With Red Rot Disease
Source: Front Microbiol. 2019 Jul 23;10:1666. doi: 10.3389/fmicb.2019.01666 (PMC6664831; doi:10.3389/fmicb.2019.01666)

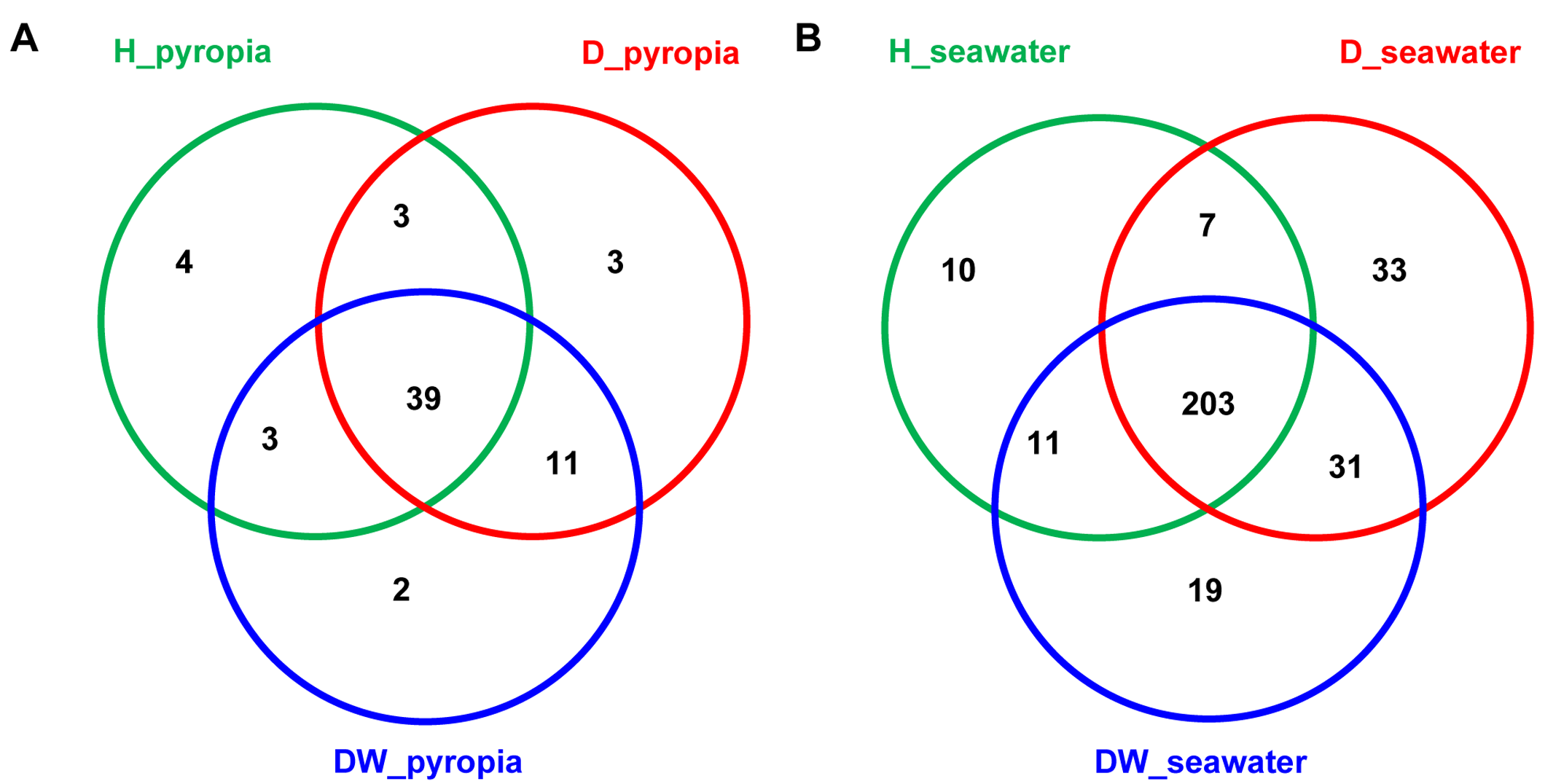

Supplement: FIGURE S1 — Venn diagrams showing the total number of OTUs and the number of shared OTUs across all Pyropia yezoensis (A) and seawater (B) datasets. [file Image_1.TIF]

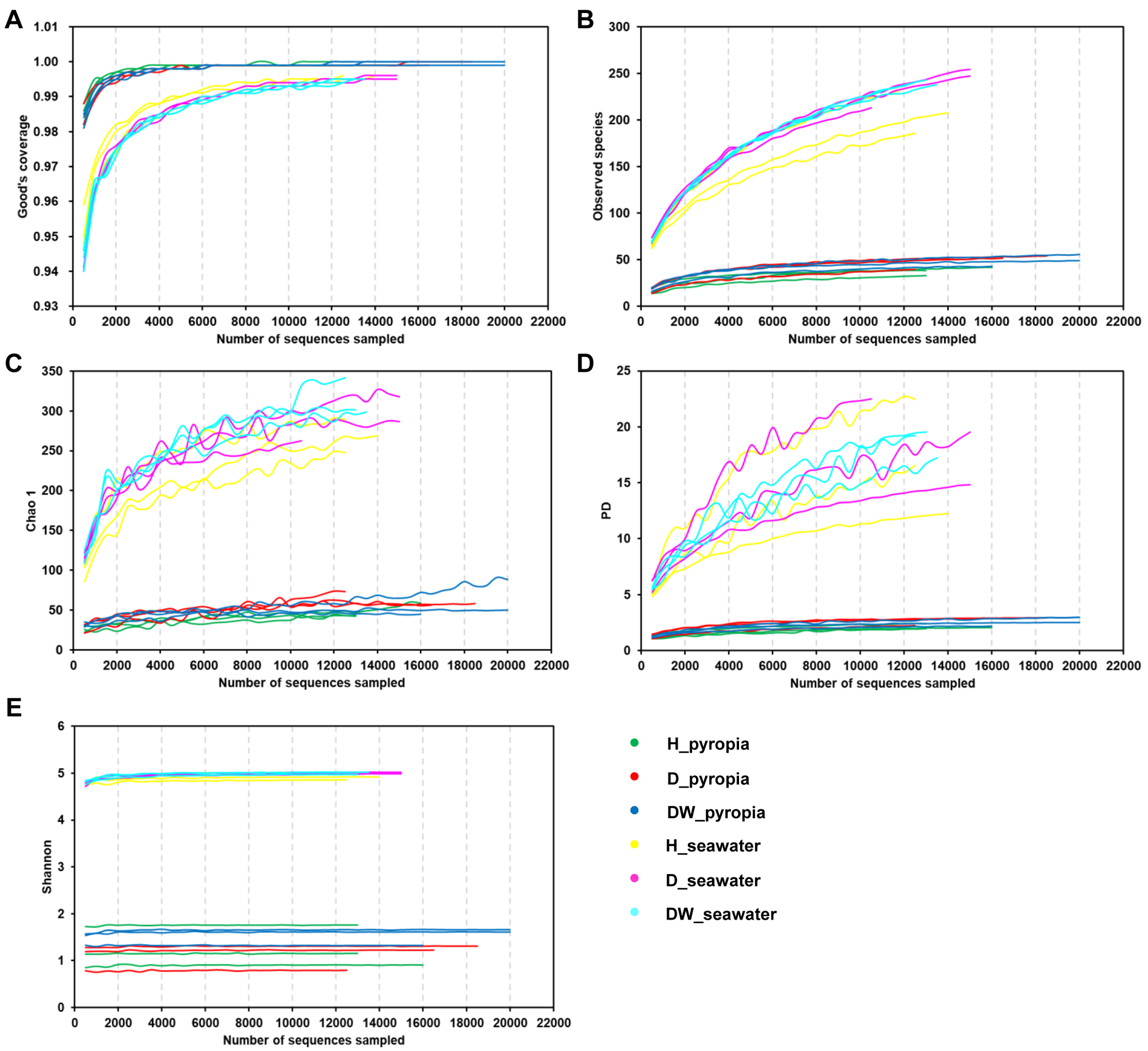

Supplement: FIGURE S2 — Rarefaction curves of Pyropia yezoensis and seawater datasets by (A) Good’s coverage, (B) Observed species, (C) Chao1, (D) PD, and (E) Shannon with OTU being defined at 97% sequence similarity. [file Image_2.TIF]

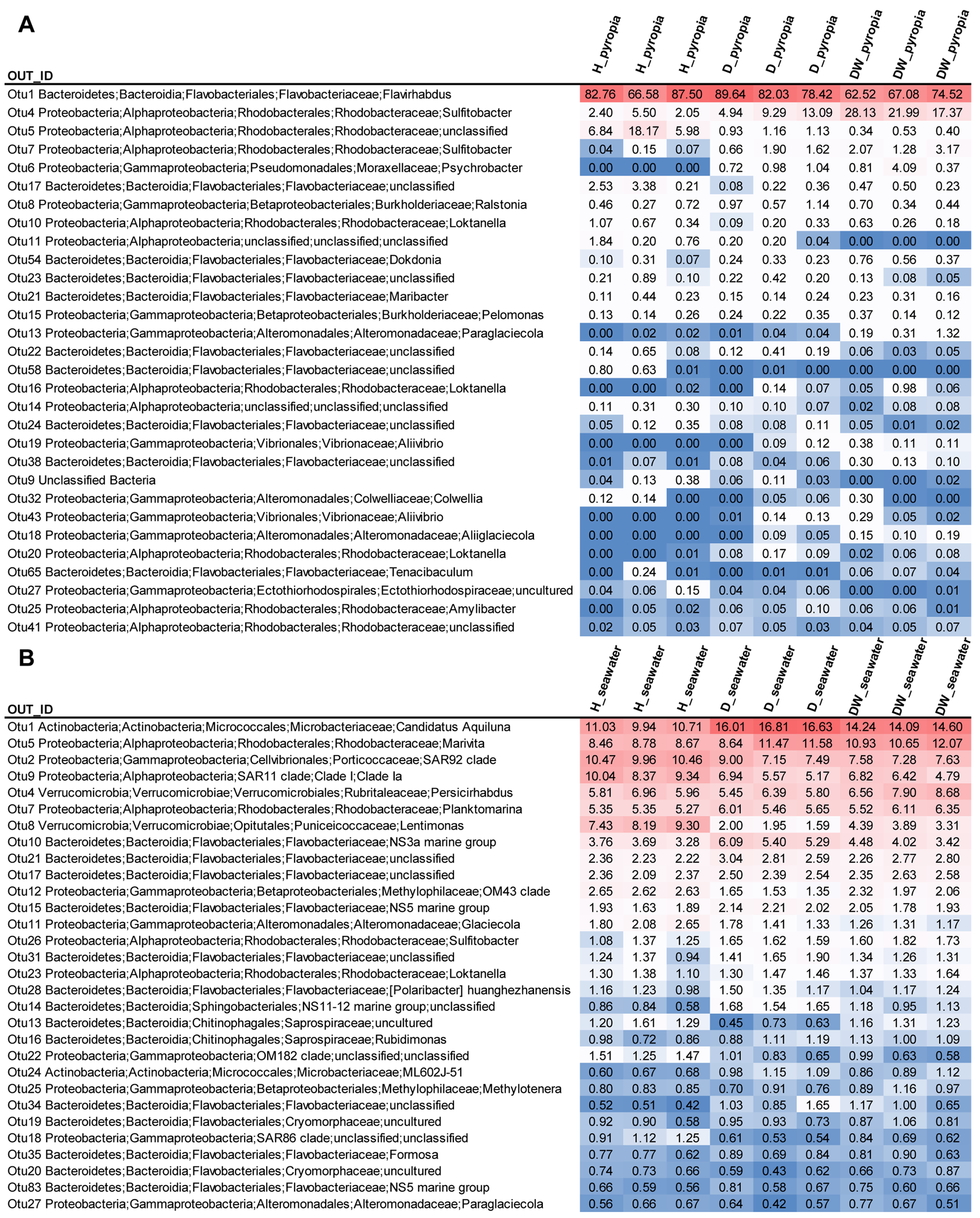

Supplement: FIGURE S3 — Distribution of the most abundant 30 OTUs across the Pyropia yezoensis (A) and seawater (B) datasets. Colors represent the relative abundance of each OTU. [file Image_3.TIF]
